# Evidence Synthesis International (ESI): Position Statement

**DOI:** 10.1186/s13643-020-01415-5

**Published:** 2020-07-10

**Authors:** David Gough, Phil Davies, Gro Jamtvedt, Etienne Langlois, Julia Littell, Tamara Lotfi, Edoardo Masset, Tracy Merlin, Andrew S. Pullin, Merel Ritskes-Hoitinga, John-Arne Røttingen, Emily Sena, Ruth Stewart, David Tovey, Howard White, Jennifer Yost, Hans Lund, Jeremy Grimshaw

**Affiliations:** 1 EPPI-Centre, University College London, London, UK; 2 Oxford Evidentia Ltd, Oxford, UK; 3 Oslo Metropolitan University, Oslo, Norway; 4 Partnership for Maternal, Newborn and Child Health, World Health Organization, Geneva, Switzerland; 5 Bryn Mawr College, Bryn Mawr, USA; 6 School of Medicine, American University of Beirut, Beirut, Lebanon; 7 Centre of Excellence for Development Impact and Learning, London International Development Centre, London, UK; 8 School of Public Health, University of Adelaide, Adelaide, Australia; 9 CEE UK Centre, Bangor University, Bangor, UK; 10 SYRCLE, Department for Health Evidence, Radboud University Medical Center, Nijmegen, Netherlands; 11 Research Council of Norway, Oslo, Norway; 12 Centre for Clinical Brain Sciences, University of Edinburgh, Edinburgh, UK; 13 Africa Centre for Evidence, University of Johannesburg, Johannesburg, South Africa; 14 Cochrane Collaboration, Oxford, UK; 15 Campbell Collaboration, Oslo, Norway; 16 Louise Fitzpatrick College of Nursing, Villanova University, Villanova, USA; 17 Centre for Evidence-based Practice, Western Norway University of Applied Sciences, Bergen, Norway; 18 Clinical Epidemiology Program, Ottawa Hospital Research Institute, Ottawa, Canada

## Abstract

This paper is the initial Position Statement of Evidence Synthesis International, a new partnership of organizations that produce, support and use evidence synthesis around the world. The paper (i) argues for the importance of synthesis as a research exercise to clarify what is known from research evidence to inform policy, practice and personal decision making; (ii) discusses core issues for research synthesis such as the role of research evidence in decision making, the role of perspectives, participation and democracy in research and synthesis as a core component of evidence ecosystems; (iii) argues for 9 core principles for ESI on the nature and role of research synthesis; and (iv) lists the 5 main goals of ESI as a coordinating partnership for promoting and enabling the production and use of research synthesis.

## Introduction

Evidence synthesis uses formal explicit rigorous methods to bring together the findings of studies already completed and to provide an account of the totality of what is known from that pre-existing research. Evidence synthesis clarifies what is known and not known about a research question. It uses research methods to provide a statement about an evidence base. It is therefore a crucial step in the use of research findings in personal and public decision making.

Evidence Synthesis International (ESI) is a new partnership of individual and umbrella organizations that produce, support and use evidence synthesis around the world. Our scope is broad, spanning education to human health, and environmental management to international development. We provide a global hub where evidence synthesis organizations meet to build and share capacities, resources and guidance, and enhance and advocate for the synthesis and use of research evidence in policy and practice decision making in all areas of human enterprise.

ESI was established following an initial exploratory meeting held alongside the HTAi (Health Technology Assessment International) annual meeting in Oslo in June 2015 and formally launched at the What Works Global Summit in London in September 2016. A foundational activity of the partnership was to develop a position statement on the shared principles and aims of ESI. This paper provides a background statement on the nature of evidence synthesis and a position statement on ESI’s principles and main areas of work. The ESI website [1] provides information on membership and governance of the initiative. The background issues and the position statement and ESI’s work will develop over time as it responds to developments in evidence synthesis and its use across the world.

## Background

### Evidence synthesis

Research can be defined as critical social enquiry for public use [2]. Research evidence is one of many factors that can inform policy, practice and individual decision making by a variety of stakeholders across all sectors of human enterprise. Prior research should also inform priorities for future research. Reliable research evidence is produced through methods that are rigorous, transparent and accountable. The bringing together of the findings of existing research studies to synthesize the totality of what is known should also use rigorous, transparent and accountable research methods.

Systematic review is a broad term to cover a range of research methods to provide rigorous and transparent reviews of research. This may be describing what research has been undertaken in relation to a research question (mapping) and/or reviewing what is known from the findings of that research (synthesis to clarify the evidence base). ESI uses the term ‘synthesis’ in its name to cover this range of systematic review methods.

Evidence synthesis can be defined as the review of what is known from existing research using systematic and explicit methods in order to clarify the evidence base and is the main focus of this journal. The research questions addressed and the methods used by such syntheses vary considerably, but they are all based on the principles of rigour and transparency. When reviews of research evidence are based on expert advice, expert panels and unsystematic methods, then the basis for the claims made by the reviews are uncertain. If reviews are not explicit and transparent in reporting their methods of review, then one cannot assess whether they have used rigorous methods that can justify the findings that they report.

A number of different organizations and collaborations have developed over the last 25 years to work on the synthesis of research in different topic areas, such as, for example:

- Effectiveness of health care interventions (Cochrane Collaboration)
- Policy and public funding recommendations in health (International Network of Agencies for Health Technology Assessment (INAHTA, WHO)
- Clinical practice recommendations in health (Guidelines International Network (GIN))
- Effectiveness of social interventions in crime, education, social care and international development (The Campbell Collaboration).
- Environmental management and sustainability (Collaboration for Environmental Evidence (CEE)).
- Preclinical and toxicological (animal) studies (Systematic Review Center for Laboratory Experimentation (SYRCLE), Collaborative Approach to Meta-Analysis and Review of Animal Data from Experimental Studies (CAMARADES)).
- Global development and health (3ie, WHO Alliance for Health Policy and Systems Research)

Other organizations have contributed to developing methods or building capacity to conduct or use evidence synthesis. For example, The Global Evidence Synthesis Initiative (GESI) aims to build capacity to conduct and use evidence syntheses in low- and middle-income country (LMIC) settings.

Different users of research with varying perspectives (values, priorities, and ideological and theoretical assumptions) may have different questions they wish research to address. This user- and question-led approach has led to a diversity of approaches, paradigms and methods in primary research, ranging from questions (and associated methods) for the study of impact of interventions to questions of prevalence, process and meaning. This user- and question-led approach is also seen in evidence synthesis with an increasing range of single and multi-component methods including statistical empirical and conceptual synthesis.

All forms of evidence synthesis are research at a ‘meta’ level as they are grounded in the analysis of pre-existing research. These synthesis methods can address a wide range of research questions including the prevalence of different phenomena, the evaluation of the impact of an intervention, the processes by which an intervention has an impact and the meaning of different experiences for people [3]. This variation in questions and methods of review results in variations in methods of synthesis and in the types of studies included in those syntheses. Included studies can range from large quantitative a priori experimental studies testing hypotheses to small scale in-depth qualitative iterative research developing theory. ‘Evidence’ could also include large scale administrative data and local feedback and monitoring data. Evidence syntheses also vary in the breadth of question and depth of analysis and how rapidly they are undertaken. They thus also vary in the extent of ‘work done’ by a synthesis and the extent of a ‘research problem’ that a review attempts or manages to address [3].

Systematic maps of research activity and synthesis of research findings can have many uses including to help prioritize research needs, encourage quality and completeness of execution and reporting of research, avoid duplication and reduce wastage of research effort [4]. They can identify research gaps that new research can fill, and they can create demand for primary research that is fit-for-purpose in terms of quality, relevance and reporting standards.

As with all research, there needs to be clarity about how rigorous and transparent research needs to be in order to make justifiable evidence claims. In other words, what are the standards of quality, rigour and relevance that should be required for different types of evidence synthesis? There has therefore been the development of standards for the execution and reporting of evidence using several types of synthesis including, for example:

- MECIR (Cochrane) [5] and MECCIR (Campbell Collaboration) [6] standards for the conduct and reporting of reviews
- PRISMA Preferred Reporting Items for Systematic Reviews and Meta-Analyses [7]
- RAMESES reporting standards for realist synthesis and meta narrative reviews [8]
- ROSES Reporting standards for Systematic Evidence Syntheses in environmental research [9]
- AMSTAR measurement tool to assess the methodological quality of systematic reviews [10]
- ROBIS tool for assessing the risk of bias in systematic reviews [11]

These examples of tools to appraise or to report systematic reviews have mostly been developed for reviews of the evidence on the effectiveness of health interventions. There is however a much wider range of review methods. It is important to have plurality in methods but the lack of agreed terminology can limit methodological collaboration within and across disciplines.

### Evidence informed decisions

Many factors may influence how a policy, practice or personal decision is made. If research evidence is used to inform a decision, then evidence synthesis can provide an understanding of the whole of an evidence base, increase certainty regarding the findings from the evidence base and explore and resolve inconsistencies in the evidence base. The findings of a single or small subgroup of studies may not represent a full and accurate picture of what is known from all available research.

The nature of the research evidence can vary considerably. Weiss, for example, distinguished between findings where empirical research findings were used instrumentally to inform decision making and new theories or concepts enlightened the thinking behind decision making [12]. Weiss distinguished both of these from the symbolic use of research where findings are ‘cherry picked’ to support positions taken for other reasons [12]. Evidence syntheses can help protect against such cherry picking evidence as they systematically identify and assess all evidence on a given research question.

Research findings require interpretation to inform decision making, and this may require the use of further types of information. The processes of interpretation and use of other forms of evidence can also be made formal explicit and accountable (and themselves be subject to research).

There is a developing interest in methods for enabling the dissemination and use of recommendations and guidelines. This includes the development of a range of tools to help clarify the process of moving from evidence to decision making, including for example:

- GRADE (Grading of Recommendations Assessment, Development and Evaluation) [13]: a system for grading the quality of evidence and strength of recommendations arising from the synthesis of impact intervention studies used by many organizations concerned with the use of health research.
- CERQual (Confidence in the Evidence from Reviews of Qualitative research): a transparent method for assessing the confidence of evidence from reviews of qualitative research [14].
- DECIDE (Tools for decision making and dissemination under development) project to develop and evaluate strategies for disseminating and supporting the uptake of guidelines by key decision makers in clinical practice [15].
- The INAHTA Product Type (IPT) Mark system which allows multi-lingual recognition and classification of different health technology assessment products, including HTA reports, mini-HTAs and rapid reviews [16].

These strategies and methods to enable the use of research evidence raise issues about the applicability of research in different contexts and feedback on how research questions are framed and relate to user needs. There is a two-way interaction between users’ needs (demand or ‘pull’) for research evidence and the production (‘push’) of relevant research findings.

### Perspectives, participation and democracy in research

The methods, standards, capacity and infrastructure available for evidence synthesis and its use raise not only technical methodological issues but also broader concerns about societal engagement with research. This includes power and agency that are reflected in questions about who should set research agendas, who should be involved in evidence synthesis and whether the scope, data, analysis, findings and recommendations sufficiently consider equity issues. The implications of omitting certain groups in society from research can lead to implications for resource allocation and perpetuate cycles of inequality [17–20]. Research and research use are not value-free and are dependent on the perspectives of those involved—their values, priorities and ideological and theoretical assumptions. Formal systems for both evidence synthesis and use are likely to vary in the perspectives they take, the decisions they are making and the research and other factors that they wish to consider. Within these systems, research issues of interest or importance may vary, between, for example, policy makers, practitioners, users of services and other individuals and groups within and between nations [21]. Specifying the mechanisms for generating and using evidence offers the potential for clarifying these differences and to determine what is, and is not, shared in perspective, approach and method. ESI’s own principles are listed later in this paper including its aim to be an open democratic learning organization. In essence, more explicit methods for evidence synthesis may benefit the development of technical systems for clarifying what is known from research and inform their quality and facilitate fit-for-purpose application. They can also increase transparency about how research is used and the perspectives and values being applied and how stakeholders are engaged in that debate. The technical processes of synthesis thus provide a basis through which there can be democratic engagement in evidence synthesis and use [22].

### Synthesis as a core component of evidence ecosystems

**Fig. 1:**
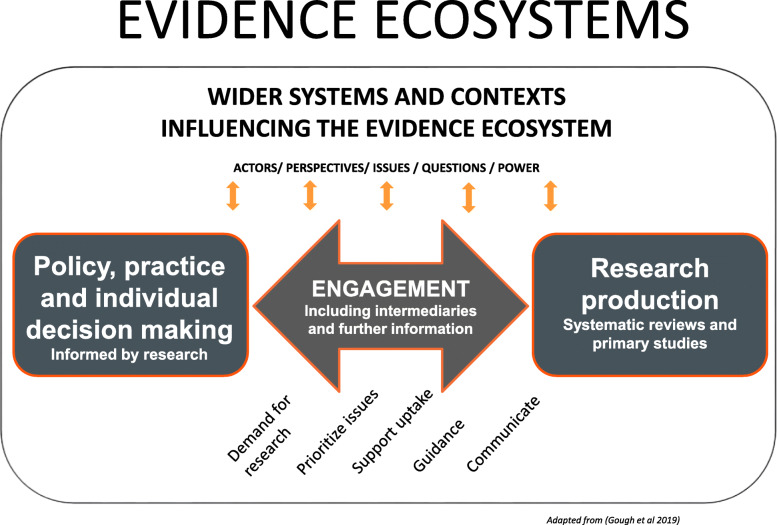
Evidence ecosystems

Research evidence is not produced in isolation. There is a two-way relationship between research being produced and it being requested and used [12]. This can be seen as an evidence ecosystem of research production and use that exists within a wider system of different perspectives, organizations and powers within society [3, 23]. Figure 1 [3] illustrates some of the main components of such a system with decisions being made, research being produced, the dynamic interaction between such demand and production, research and other forms of information involved in interpreting research findings and the wider contexts and forces within which such evidence ecosystems function.

If research is not undertaken, not undertaken well, is not relevant in focus for potential users of research or is not synthesized to provide an account of what is known from all available research, then the evidence is not likely to be used appropriately or effectively. Evidence is usually used to some extent and so evidence ecosystems exist even if they do not always function effectively. Synthesis is a key component of evidence ecosystems in creating and providing statements of what is known from research.

Taking a systems approach can also help in planning the production and use of research. Different stakeholder and decision makers can be consulted about their priorities for research evidence. Synthesis can clarify what is known and not known and how further primary research can inform such users’ needs. Synthesis is therefore key in informing the prioritization, methods and use of primary research.

Similarly, strategic choices can also be made about the balance of effort and investment in different parts of an ecosystem and help identify waste in research use and research production. Is there, for example, an appropriate balance between investments in the production of primary research and the synthesis of that research?

### Inviting global dialogue and collaboration in evidence synthesis and evidence use

As many organizations in different fields in different parts of the world are developing methods and infrastructure for evidence synthesis and use, the opportunity arises for shared learning and joint work. This could involve working together on principles, standards, methods, funding, teaching infrastructure and advocacy for synthesis. It could enable joint work, reduce duplication, optimize investment of effort, clarify variation in approaches and encourage innovation and development. ESI is a membership organization made up of organizations around the world that produce, support and use evidence syntheses. Some of the members of Evidence Synthesis International are umbrella organizations or networks in their own right. They have individual organizational members with a common purpose. Mention has already been made of GESI. This started as an initiative of the Alliance for Health Systems and Policy Research, the Campbell Collaboration, Cochrane, the EPPI-Centre and 3ie and now has a Secretariat, more partners and a network across 47 centres in 25 LMICs with a common concern to build capacity for conduct and use of evidence syntheses. It has also ensured the inclusion of evidence researchers from LMICs in the update of the Cochrane Handbook in 2018.

Another example is the International Network of Agencies for Health Technology Assessment (INAHTA) which represents 50 not-for-profit health technology assessment agencies in 31 countries.

The aim of creating the ESI collaboration and this position statement is to provide a broader space and profile for these and other initiatives to work together to achieve our common aims. ESI respects the remit and responsibilities of these individual and umbrella organizations within their particular sectors (e.g. health, education, environment) and in no way intends to duplicate their activities. Rather, the focus of ESI is to advocate for the importance of evidence synthesis *across* different sectors and to act as a hub for *cross-sectoral* collaboration.

Such a macro-level collaboration between research synthesis entities can advocate for and develop higher expectations internationally for better methods, standards, investment, capacity and infrastructure in evidence synthesis and its use. One aspiration, for example, could be for a minimum percentage of research investment to be used for the synthesis of research. Another could be for agreed reporting standards across different topic and methods areas, as well as improved liaison to reduce duplication of effort. Another could be for all public bodies to have explicit policies on processes for the use of synthesized evidence in decision making. The work on evidence synthesis and use has the potential for an enormous positive impact on societies and there is much scope for greater engagement with, for example, international organizations involved in all areas of social and environmental policy. This position statement provides a starting point for such collaboration and the work of ESI.

### Producing an ESI position statement

Systematic reviews and research synthesis (and their importance for the production and use of research findings) are central to the aims and values of ESI. The organization has therefore worked to create a set of principles for its work and goals for how this would be achieved. These principles were originally drafted by the initial instigators of ESI and then refined and developed by the authors of this paper as Members of the ESI Steering Group, the Board of Directors and the Secretariat.

## Position statement

### Principles

These are the shared principles underlying the work of ESI:

1. Evidence synthesis is a vital component in: (i) identifying what is known and not known from research; and (ii) using such research evidence to make well informed decisions.

Evidence from appropriate research can be critical for individuals, groups and society in making informed decisions. In some cases, there is a lack of appropriate research evidence. Evidence synthesis can make this explicit and inform future primary research. Evidence synthesis provides a means to clarify what we know, what we do not know and what more we need to know. It can enable more strategic thinking about research use, research needs and research policy and planning.

2. Evidence synthesis should follow question-led formal methods that are rigorous, transparent and accountable.

Research evidence may be able to inform and assist decision making by different groups and individuals in societies. For this to happen in an accountable way, there is a need for fit-for-purpose methods of evidence synthesis (bringing together what we know and how we know it) applied ethically, rigorously and transparently and completely reported. Individual research studies make up such evidence bases but should not be relied upon individually without situating them within the wider evidence base identified through systematic evidence synthesis.

Evidence synthesis is a form of research and so should conform to the expectations of research including the use of formal transparent methods. All research and its interpretation are driven by the questions being asked and their inherent perspectives (values, priorities, and ideological and theoretical assumptions) and so these should also be transparent. Evidence synthesis that is not rigorous, relevant and explicit and complete in method and reporting (such as expert literature reviews) or is driven by unspecified vested interests does not conform to the expectations of research. Synthesis that does not meet such appropriate evidence standards is not sufficient for making justifiable evidence claims and so should not be relied upon [24]. There may not always be agreement about what specific levels of rigour and explicitness of methods are considered sufficient for an evidence synthesis to make a justifiable evidence claim to inform decision making. Evidence syntheses are not homogeneous in their type or breadth of question, methods, included studies, depth of analysis, rigour and ‘work done’ by a review [3]. Variation in the specific criteria for making distinctions between types of syntheses and their methods and their being ‘borderline’ cases of how systematic a synthesis may be does not undermine the argument for all research, including syntheses, to be rigorous, relevant and with explicit and accountable reporting of methods.

3. There should be formal processes to inform the transparent interpretation and use of the findings of evidence synthesis.

In addition to methods for the synthesis of research, transparent processes are also required for the interpretation and application of evidence synthesis when making policy, practice and personal decisions. In order to enable the accountable use of evidence synthesis, the interpretation and application of that evidence (alongside the social values, contexts, and all the other evidence and wider factors that inform decision making) should be consistent and transparent.

4. Research paradigms, perspectives, questions, methods, data, analysis and interpretation and application may vary but all can be useful (fit for purpose) in different ways to different stakeholders and under different circumstances.

Individuals and organizations may differ in their perspectives (values, priorities, and ideological and theoretical assumptions) and in the type of research questions considered appropriate, the methods used to address such questions and the purposes to which the research is put. Such variation exists in both primary research and research synthesis but does not undermine the logic and rationale of the production of evidence synthesis. Research evidence is constructed and interpreted within social perspectives. The transparent formal processes of principle 2 help clarify the perspectives being applied. Such transparency can also help clarify the way that different perspectives in society (including members of the public and users of services) participate in research production and use.

5. Interpretation and use of evidence synthesis may inform decision making in various ways, for example, through providing empirical data (instrumental effect) or through ways of conceptualizing and understanding issues (enlightenment effect).

The application of evidence synthesis should be logical (within the perspectives within which it is being used), notwithstanding the nature of that evidence or whether the impact is empirical or conceptual. Evidence used selectively to support decisions made on other grounds is a symbolic use of evidence.

6. Interpretation and use of evidence synthesis to inform decision making often involves the use of other evidence and information beyond research evidence.

Evidence synthesis informs judgements made in decision making; it does not mechanically determine decision making. Decisions may be based on research evidence combined with many other factors such as values, tacit knowledge, available resources and local conditions and contexts. Transparency in the synthesis and use of research makes clear how different perspectives, values and types of information inform decision making.

7. The ability of evidence synthesis to provide rigorous evidence to inform the decisions of individuals, groups and societies is advanced by the study and development of evidence synthesis methodologies and capacity strengthening for the production and use of evidence synthesis.

Methods of evidence synthesis and processes for research use are not fixed; they are continually developing and require ongoing learning and training. There is a need for the ongoing development of methods to both undertake and study the synthesis and use of research.

8. ESI will specify the social values it follows in terms of both research synthesis and research use

These social values include:

- Ethical high-quality transparent rigorous fit-for-purpose methods of research and the avoidance of waste in research;
- Social justice, equity, social inclusion and the valuing of different perspectives in research production and its use;
- Research making an important contribution to societal decision making and access to research as a democratic societal *right.*

9. ESI will specify the principles for its own governance

These principles include developing a governance and membership structure that:

- Is a learning organization, open to different perspectives and experiences in evidence synthesis
- Is inclusive in working with those with similar aims and principles in using systematic approaches in the review and use of research evidence even if they may not in practice always fully meet those aims and principles
- Has a governance and membership structure that is transparent, open, inclusive and democratic with its principles also reflected in the constituent membership organizations
- Is open and transparent about its values and in its measures and performance in avoiding potential conflicts of interest
- Has no individual organization, or sector, role, topic or geographical focus dominating either the Board or the Sub-Committees of ESI
- Operationalizes these principles, monitors compliance and corrects any departure from them.

## Goals

### 1. Advocacy

#### 1.1 To use advocacy to achieve political and resource support to achieve the shared principles and aims of ESI

In order to achieve the aims implicit in all the principles stated in this paper, ESI will advocate for increased political and financial support for the development and use of evidence synthesis in decision making. The move from a largely expert or ‘ad hoc’ use of evidence in many political and practice decision making processes to a more structured approach (as in principle 6) will, for example, require considerable political support. Advocacy (that is, preferably, evidence-informed in approach) can help to engage such support. This might include, for example, the use of success stories from the global community of the beneficial effects of evidence-informed decision making. It may also include support for new organizations or networks for coordinating such efforts in new fields where those are not existing.

### 2. Infrastructure, policies and governance to support evidence synthesis

#### 2.1 To develop international, national and organizational policies, infrastructures, procedures, processes and resources to enable the development of systematic approaches to evidence synthesis and use.

Research evidence is an important form of information in societal decision making. The conduct and use of evidence synthesis do not only require methods and capacity; it requires policies and systems by which it can be enabled, monitored, accessed, used and evaluated and integrated into broader systems for planning and decision making. Without these, evidence may not be used or might be used in a partial way that does not represent the totality of relevant evidence (through lack of access, lack of rigour or due to non-transparent perspectives and interests). Examples include infrastructure, policies and governance for:

- Prioritizing research needs
- Undertaking systematic reviews such as systematic maps and syntheses
- Complete reporting and enabling access to such maps and syntheses
- Interpretations and application of research evidence including structured processes for advice and guidance
- Understanding and use of overarching approaches to how evidence is required, produced, understood and used at individual, group, national and international levels
- Providing access to approaches and models and sharing lessons learnt;
- Enabling tools, resources, and software to be accessible and usable by researchers globally including those with less resources and capacity

### 3. Funding

#### 3.1 To enable funding to support sustainable business models for the shared principles and aims of ESI to be achieved and developed.

The aims and principles cannot be achieved without adequate resourcing. This may be achieved through additional funding of evidence ecosystems or a rebalancing of strategy and investment across such ecosystems (such as the balance between the funding of primary research and synthesis of that research). ESI’s aim is to attract funding for its members to achieve ESI’s goals. ESI will also require some funds in order to achieve its coordinating function.

### 4. Methodology: procedural, methodological and technical development of synthesis methods

#### 4.1 To develop and share standards and terminology to help guide, monitor and raise expectations for quality and relevance of procedures and methods for evidence synthesis and use.

The development of joint methodological standards and terminology may reduce some duplication of effort whilst still allowing plurality in methods and perspectives and the scope for further innovation and development. Some standards may be shared whilst others may be specific to the topic, sector, stakeholder or perspective.

#### 4.2 To develop and share fit-for-purpose procedures, methods and tools that allow for diversity in purpose and execution and development within the shared principles of evidence synthesis and use.

The joint development of evidence synthesis methods may reduce duplication of effort whilst still allowing plurality and the scope for further innovation and methodological development. Evidence synthesis and use is a relatively new area of research and has developed most for examining evidence on the impact of interventions in health. As further questions, topics and perspectives from a wider group of stakeholders are addressed, new methods and approaches will continue to develop within a culture that encourages innovation and freedom to experiment.

### 5. Learning and teaching

#### 5.1 To develop academic learning and capacity in evidence synthesis and use.

Undertaking the various forms of evidence synthesis requires specific skills, and these are changing as new forms of synthesis are developed. There is a need to learn from new areas and to develop capacity to undertake and understand different approaches to evidence synthesis for those involved in all levels of learning across all disciplines and topics. This might include ESI members working on mentorship and skills exchange programmes to enhance the quality of and access to work in this area internationally. It might also involve ESI member organizations collaborating to deliver training and capacity development in countries where evidence synthesis is emerging.

#### 5.2 To develop broader societal learning and capacity in evidence synthesis and use.

The ability to understand the aims, approach and results of evidence synthesis can assist various stakeholders in their ability to commission and make use of evidence synthesis. There is also a broader societal role for knowledge of synthesis to be available to citizens in order to be able to facilitate engagement in democratic debates involving evidence. Those commissioning, conducting and using evidence synthesis can benefit from learning about how to engage with the variation in stakeholder needs and the legitimacy of evidence within decision making systems. For example, evidence synthesis researchers from the environmental field developed a five-step approach to engage stakeholders in evidence synthesis [25]. Similarly, Cochrane launched an international network for public involvement and engagement in health and social care research [26]. INAHTA has member agencies, such as NICE [27] and the (CADTH) [28]) that closely involve patients and citizens in the development and deliberations concerning evidence syntheses.

## Summary

ESI has been founded to advocate for the importance of and to develop the capacity for undertaking research synthesis. In doing so, it is important to specify the principles and the goals of ESI. More broadly, the position statement can help develop a debate and further our thinking about what are the fundamental principles of research synthesis in research and in wider society.

The position statement of principles and goals developed by ESI are inclusive and yet respectful of the variation in approach, perspective and responsibilities of its members. They have been developed to demonstrate our shared understanding of the value of evidence synthesis to society and our vision to have this value reflected in the policies, practices and resources utilized in all sectors and by all stakeholders internationally. The principles and goals will evolve over time as society’s needs change and as our sophistication and understanding of research synthesis develops.

## Data Availability

Not applicable

## Abbreviations

AMSTAR: A MeaSurement Tool to Assess systematic Reviews
3ie: International Initiative for Impact Evaluation
CADTH: Canadian Agency for Drugs and Technologies in Health
CERQual: Confidence in the Evidence from Reviews of Qualitative research
CAMARADES: Collaborative Approach to Meta-Analysis and Review of Animal Data from Experimental Studies
CEE: Collaboration for Environmental Evidence
DECIDE: Developing and Evaluating Communication Strategies to support Informed Decision and practice based on Evidence
EPPI-Centre: Evidence for Policy and Practice Information and Coordinating Centre
ESI: Evidence Synthesis International
GESI: Global Evidence Synthesis Initiative
GRADE: Grading of Recommendations Assessment, Development and Evaluation
GIN: Guidelines International Network
HTAi: Health Technology Assessment International
INAHTA: International Network of Agencies for Health Technology Assessment
LMIC: Lower- and middle-income countries
MECIR: Methodological Expectations of Cochrane Intervention Reviews
MECCIR: M*ethodological Expectations of Campbell Collaboration Intervention Reviews*
NICE: National Institute for Health and Care Excellence
PRISMA: Preferred Reporting Items for Systematic Reviews and Meta-Analyses
RAMESES: Realist And MEta-narrative Evidence Syntheses
ROSES: RepOrting standards for Systematic Evidence Syntheses in environmental research
ROBIS: Risk of bias in systematic reviews
SYRCLE: Systematic Review Center for Laboratory animal Experimentation
WHO: World Health Organization
